# Asthmatic Eosinophils Alter the Gene Expression of Extracellular Matrix Proteins in Airway Smooth Muscle Cells and Pulmonary Fibroblasts

**DOI:** 10.3390/ijms23084086

**Published:** 2022-04-07

**Authors:** Ieva Janulaityte, Andrius Januskevicius, Airidas Rimkunas, Jolita Palacionyte, Astra Vitkauskiene, Kestutis Malakauskas

**Affiliations:** 1 Laboratory of Pulmonology, Department of Pulmonology, Lithuanian University of Health Sciences, LT-44307 Kaunas, Lithuania; andrius.januskevicius@lsmuni.lt (A.J.); airidas.rimkunas@lsmuni.lt (A.R.); kestutis.malakauskas@lsmuni.lt (K.M.); 2 Department of Pulmonology, Lithuanian University of Health Sciences, LT-44307 Kaunas, Lithuania; jolita.palacionyte@lsmuni.lt; 3 Department of Laboratory Medicine, Lithuanian University of Health Sciences, LT-44307 Kaunas, Lithuania; astra.vitkauskiene@lsmuni.lt

**Keywords:** asthma, allergy, eosinophil, extracellular matrix proteins, TGF-β signaling pathway, airway smooth muscle cells, pulmonary fibroblasts, airway remodeling

## Abstract

The impaired production of extracellular matrix (ECM) proteins by airway smooth muscle cells (ASMC) and pulmonary fibroblasts (PF) is a part of airway remodeling in asthma. This process might be influenced by eosinophils that migrate to the airway and abundantly secrete various cytokines, including TGF-β. We aimed to investigate the effect of asthmatic eosinophils on the gene expression of ECM proteins in ASMC and PF. A total of 34 study subjects were recruited: 14 with allergic asthma (AA), 9 with severe non-allergic eosinophilic asthma (SNEA), and 11 healthy subjects (HS). All AA patients underwent bronchial allergen challenge with *D. pteronyssinus*. The peripheral blood eosinophils were isolated using high-density centrifugation and magnetic separation. The individual cell cultures were made using hTERT ASMC and MRC-5 cell lines and the subjects’ eosinophils. The gene expression of ECM and the TGF-β signaling pathway was analyzed using qRT-PCR. We found that asthmatic eosinophils significantly promoted collagen I, fibronectin, versican, tenascin C, decorin, vitronectin, periostin, vimentin, MMP-9, ADAM33, TIMP-1, and TIMP-2 gene expression in ASMC and collagen I, collagen III, fibronectin, elastin, decorin, MMP-2, and TIMP-2 gene expression in PF compared with the HS eosinophil effect. The asthmatic eosinophils significantly increased the gene expression of several canonical and non-canonical TGF-β signaling pathway components in ASMC and PF compared with the HS eosinophil effect. The allergen-activated AA and SNEA eosinophils had a greater effect on these changes. In conclusion, asthmatic eosinophils, especially SNEA and allergen-activated eosinophils, imbalanced the gene expression of ECM proteins and their degradation-regulating proteins. These changes were associated with increased gene expression of TGF-β signaling pathway molecules in ASMC and PF.

## 1. Introduction

Thickened airway walls characterize airway remodeling in asthma due to the increased proliferation and activation of airway smooth muscle cells (ASMC) and pulmonary fibroblasts (PF), imbalanced extracellular matrix (ECM) homeostasis, and neovascularization [1]. The ECM determines the tissue architecture of the airways, thus providing mechanical stability and elastic recoil, which are essential for normal lung function. Structural changes in the airway are the pathological features contributing to the disease’s clinical manifestations, such as bronchoconstriction, wheezing, and shortness of breath. Chronic inflammation in asthma is orchestrated by the direct and indirect cross-talk of various cells. Eosinophilic asthma is characterized by blood, airway tissue, and sputum eosinophilia with high levels of TGF-β that are abundantly secreted by eosinophils and airway structural cells such as ASMC and PF [2]. It was shown that the profibrotic cytokine TGF-β participates in the main remodeling processes in asthma, including cellular and structural changes in large and small airways [3]. TGF-β is accumulated in a non-active form as the large latent complex in ECM and can regulate the synthesis and degradation of ECM components [4]. Furthermore, the matrix metalloproteinases (MMP), such as MMP-2 and MMP-9, degrade latent TGF-β complexes, thus increasing the active TGF-β form in ECM [5].

ECM proteins in the airway can be divided into two main groups: (1) basement membranes (collagen IV, perlecan, and laminins) and (2) interstitial matrices (collagens, elastin, laminins, fibronectin, tenascin, decorin, biglycan, and versican). The basement membrane is a sheet-like deposition of ECM on which epithelial cells rest. At the same time, interstitial matrices form a loose or fibril-like network that interconnects structural cell types to form three-dimensional cohesiveness and the biomechanical characteristics of the airway. Moreover, interstitial matrices serve as the platform for complex signaling, which regulates the differentiation and function of structural cells [6]. It was shown that ECM components such as collagen, elastic fibers, fibronectin, and versican are highly expressed in asthmatic airways [7]. ECM homeostasis is an essential part of development and wound healing, while the disbalance of ECM deposition and degradation results in pathological conditions for cells [8]. ECM homeostasis is regulated by MMPs that degrade ECM proteins and the tissue inhibitors of matrix metalloproteinases (TIMP), which modulate the activity of MMPs. Changes in the microenvironment cause behavioral changes in the structural airway cells. In asthma, the qualitative and quantitative changes in ECM are part of the airway remodeling processes that contribute to asthma pathogenesis [9]. 

A TGF-β signaling pathway consists of canonical (Smad-dependent) and non-canonical pathways that start when TGF-β is synthesized and bound into latent TGF-β complexes, activated in ECM by MMPs [10]. The active TGF-β binds to TGF-β receptors on the outer membrane of cells. Then, Smad2/3 and Smad4 signaling start the canonical or the non-canonical signaling pathways that result in the transcription of several cytokines, the synthesis of ECM proteins, and actin polymerization [11]. The eosinophil-promoted expression of TGF-β signaling pathway elements and the downregulation of inhibition molecules are associated with activated TGF-β signaling in cells [12,13,14]. We hypothesized that the asthmatic eosinophils affect the gene expression of ECM proteins and the activation of the TGF-β signaling pathway in ASMC and PF.

## 2. Results

### 2.1. Characteristics of the Study Population

We investigated 34 nonsmoking adults (15 men and 19 women): 14 steroid-free non-severe allergic asthma (AA) patients, 9 severe non-allergic eosinophilic asthma (SNEA) patients with a high dose of inhaled steroids, and 11 healthy subjects (HS). The demographic and clinical characteristics of the study population are presented in Table 1. The SNEA patients were significantly older than the AA and HS groups. Moreover, the degree of lung function was significantly lower in the SNEA group than in the other groups. Furthermore, the eosinophil count, immunoglobulin E (IgE) concentration, and FeNO level were significantly higher in the AA and SNEA groups compared with the HS group.

The bronchial allergen challenge with D. pteronyssinus allergen was performed on all AA patients. A significant increase in the eosinophil count and IgE concentration in the blood was observed following allergen exposure.

### 2.2. Gene Expression of ECM Proteins in Structural Airway Cells

**Figure 1:**
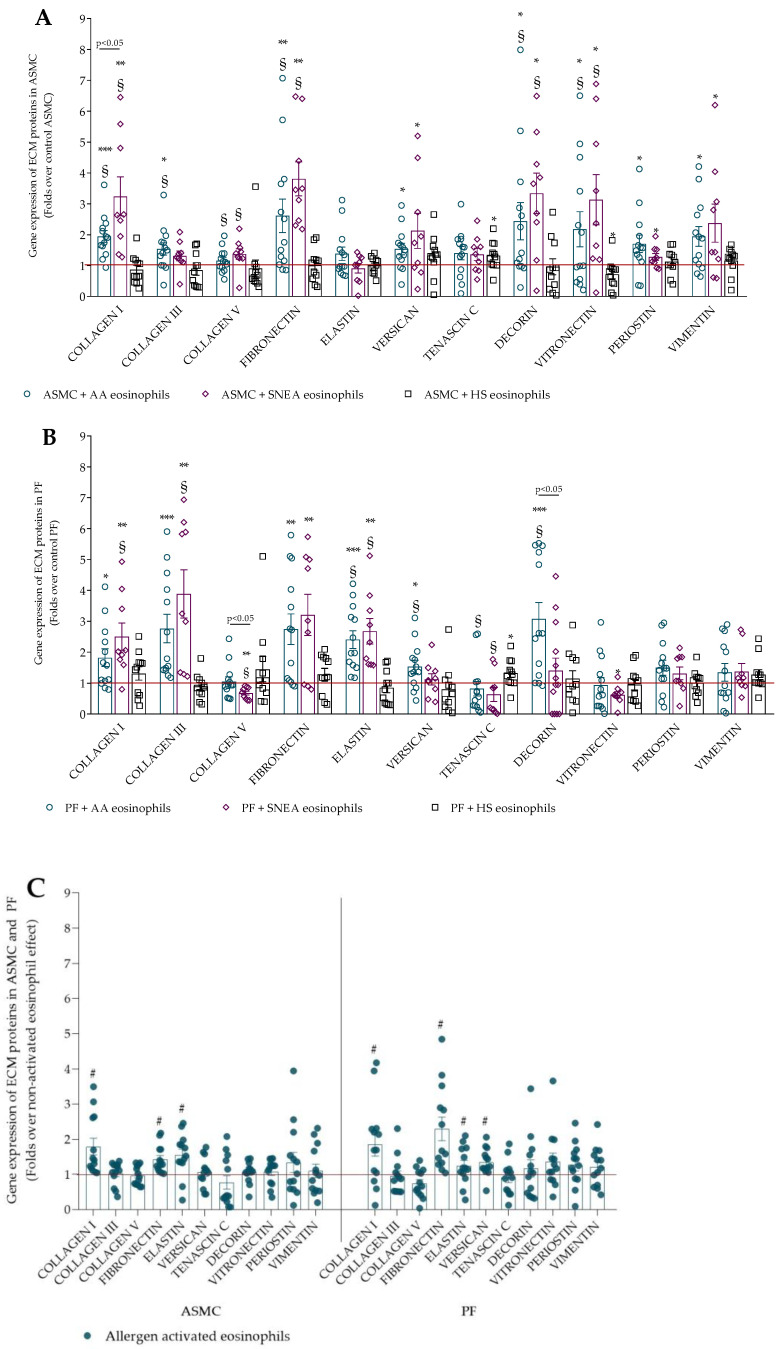
(**A**)—Gene expression of ECM proteins in ASMC; (**B**)—gene expression of ECM proteins in PF; (**C**)—gene expression of ECM proteins in ASMC and PF after incubation with allergen-activated eosinophils. Data presented as mean ± SEM, fold change over control ASMC or PF, and fold change over eosinophil effect before bronchial allergen challenge. AA—allergic asthma; ASMC—airway smooth muscle cell; HS—healthy subject; PF—pulmonary fibroblasts; SNEA—severe non-allergic eosinophilic asthma. AA *n* = 13; SNEA *n* = 9; HS *n* = 11. * *p* < 0.05 compared with control ASMC or PF; ** *p* < 0.01 compared with control ASMC or PF; *** *p* < 0.001 compared with control ASMC or PF; § *p* < 0.05 compared with HS eosinophil effect; # *p* < 0.05 compared with non-activated eosinophil effect. Statistical analysis between investigated groups—two-sided Mann–Whitney U test (independent data); two-sided Wilcoxon matched-pairs signed-rank test (dependent data); and Wilcoxon signed-rank test, which was used for gene expression analysis against control ASMC or PF and to compare changes in the effect of eosinophils on ASMC or PF before and 24 h after bronchial allergen challenge.

Several ECM proteins are associated with airway fibrosis, a part of airway remodeling in asthma. ECM proteins were selected based on the literature, which shows a significant impact on asthma pathogenesis in terms of changing cell behavior or the physiological properties of airways. We evaluated the eosinophil effect on ECM gene expression and found that AA eosinophils significantly increased collagen I, collagen III, fibronectin, vitronectin, periostin, and vimentin gene expression in ASMC compared with control ASMC (Figure 1A). Eosinophils from SNEA eosinophils significantly increased collagen I, fibronectin, versican, decorin, vitronectin, periostin, and vimentin gene expression in ASMC compared with control ASMC cells. Eosinophils from HS significantly increased tenascin C and decreased vitronectin gene expression compared with control ASMC.

Furthermore, the eosinophils from AA and SNEA patients significantly increased collagen I, collagen V, fibronectin, decorin, and vitronectin gene expression in ASMC compared with the HS eosinophils effect. Additionally, eosinophils from AA significantly increased collagen III gene expression in ASMC compared with eosinophils from HS.

The collagen I and fibronectin gene expression in ASMC was significantly higher after incubation with SNEA eosinophils than AA eosinophils. Additionally, the allergen-activated eosinophils significantly increased collagen I, fibronectin, elastin, and periostin gene expression in ASMC compared with the non-activated eosinophils (Figure 1C). 

Asthmatic eosinophils significantly increased collagen I, collagen III, fibronectin, elastin genes expression in PF compared to control PF. Additionally, AA eosinophils increased versican and decorin genes expression in PF compared to control PF. Asthmatic eosinophils significantly promoted the gene expression of collagen III, fibronectin, elastin, and decorin in PF compared with the HS eosinophils (Figure 1B). Furthermore, the gene expression of collagen V in PF was promoted only by the AA eosinophils, while the gene expression of vimentin was promoted only by SNEA eosinophils compared with the HS group. The SNEA eosinophils significantly decreased vitronectin gene expression in PF compared with the HS eosinophils. The allergen-activated eosinophils significantly promoted collagen I, fibronectin, elastin, and versican gene expression in PF compared with the non-activated eosinophils (Figure 1C). All *p* values are presented in Appendix C.

### 2.3. Gene Expression of MMPs and TIMPs in Structural Airway Cells

**Figure 2:**
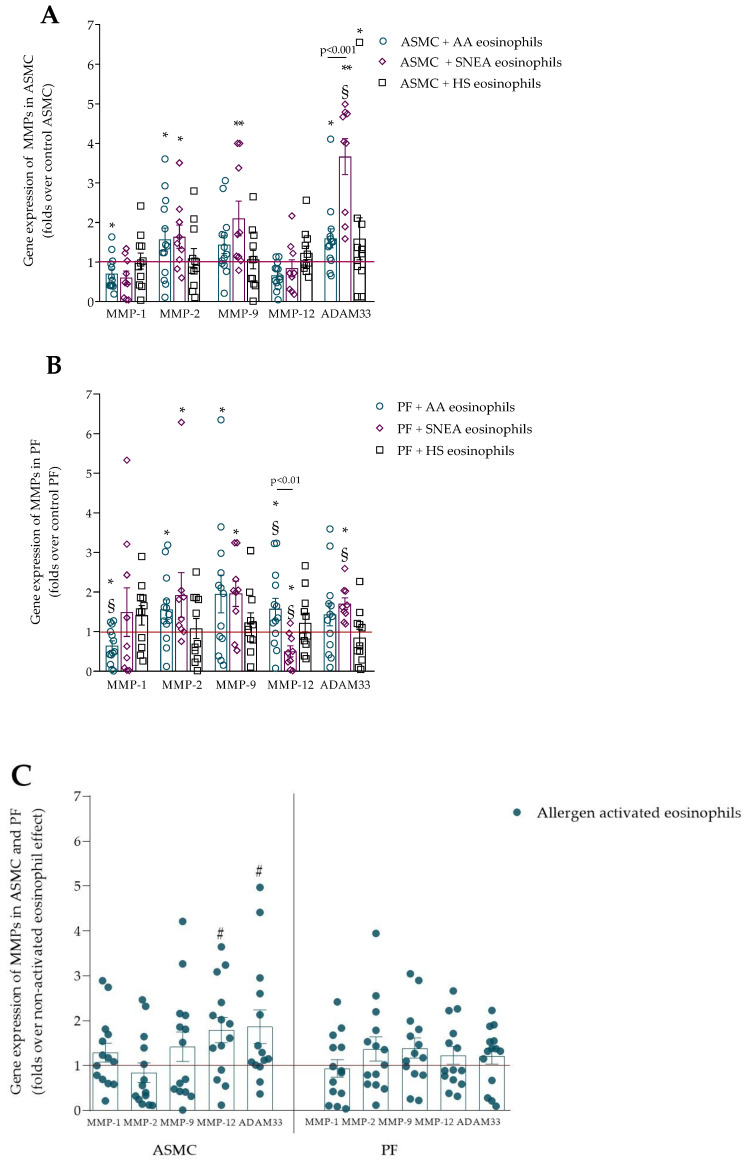
(**A**)—Gene expression of MMPs in ASMC; (**B**)—gene expression of MMPs in PF; (**C**)—gene expression of MMPs in ASMC and PF after incubation with allergen-activated eosinophils. AA—allergic asthma; ADAM33—a disintegrin and metalloprotease 33; ASMC—airway smooth muscle cell; HS—healthy subject; MMP—matrix metalloproteinase; PF—pulmonary fibroblasts; SNEA—severe non-allergic eosinophilic asthma. AA, *n* = 13; SNEA, *n* = 9; HS, *n* = 11. Data are presented as mean ± SEM, fold change over control ASMC or PF, and fold change over non-activated AA eosinophils. * *p* < 0.05 compared with control ASMC or PF cells; ** *p* < 0.01 compared with control ASMC or PF cells; § *p* < 0.05 compared with HS eosinophil effect; # *p* < 0.05 compared with non-activated eosinophil effect. Statistical analysis between investigated groups—two-sided Mann–Whitney U test (independent data); two-sided Wilcoxon matched-pairs signed-rank test (dependent data); and Wilcoxon signed-rank test, which was used for gene expression analysis against control ASMC or PF that were not incubated with eosinophils.

MMPs are a group of proteins that degrade ECM proteins. In the current study, we selected the main MMPs, including MMP-1, MMP-2, MMP-9, and MMP-12, and the newest MMP of interest—ADAM33. AA eosinophils significantly increased MMP-2 but decreased MMP-1 gene expression in ASMC compared with control ASMC (Figure 2A). SNEA eosinophils increased MMP-2 and MMP-9 gene expression in ASMC cells compared with control ASMC. Furthermore, the ADAM33 gene expression in ASMC was increased after incubation with eosinophils from all the study groups, but the SNEA eosinophils had the most significant effect on ADAM33 gene expression in PF, compared with AA and HS eosinophils. AA eosinophils significantly increased MMP-2, MMP-9, and MMP-12 but decreased MMP-1 gene expression in PF compared with control PF (Figure 2B). Additionally, the MMP-12 gene expression after incubation with AA eosinophils was significantly higher than that after incubation with HS eosinophils or SNEA eosinophils. SNEA eosinophils significantly increased MMP-2 and MMP-9 gene expression in PF compared with control PF. The ADAM33 gene expression was significantly higher after incubation with SNEA eosinophils compared with HS eosinophils and the control PF.

The gene expression of MMP-12 and ADAM33 in ASMC was significantly higher after incubation with allergen-activated eosinophils compared with non-activated eosinophils (Figure 2C). The allergen-activated eosinophil effect on the MMP expression of PF showed no significant differences compared with the non-activated eosinophil effect. 

**Figure 3:**
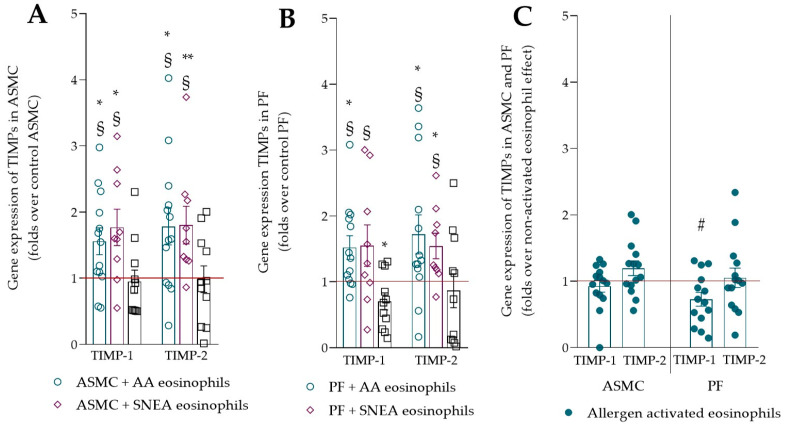
(**A**)—Gene expression of TIMPs in ASMC; (**B**)—gene expression of TIMPs in PF; (**C**)—gene expression of TIMPs in ASMC and PF after incubation with allergen-activated eosinophils. AA—allergic asthma; ASMC—airway smooth muscle cell; HS—healthy subject; PF—pulmonary fibroblasts; SNEA—severe non-allergic eosinophilic asthma; TIMP-1—tissue inhibitor of metalloproteinases 1; TIMP-2—tissue inhibitor of metalloproteinases 2. AA, *n* = 13; SNEA, *n* = 9; HS, *n* = 11. Data are presented as mean ± SEM, fold change over control ASMC or PF, and fold change over non-activated AA eosinophils. * *p* < 0.05 compared with control ASMC or PF; ** *p* < 0.01 compared with control ASMC or PF; § *p* < 0.05 compared with HS eosinophil effect; # *p* < 0.05 compared with non-activated eosinophil effect. Statistical analysis between investigated groups—two-sided Mann–Whitney U test (independent data); two-sided Wilcoxon matched-pairs signed-rank test (dependent data); and Wilcoxon signed-rank test, which was used for gene expression analysis against control ASMC or PF that were not incubated with eosinophils.

The gene expression of TIMP-1 and TIMP-2 increased in ASMC and PF after incubation with asthmatic eosinophils compared with HS eosinophils and control ASMC and PF (Figure 3A,B). Eosinophils from HS significantly decreased TIMP-1 gene expression in PF compared with control PF. Allergen-activated eosinophils significantly downregulated the gene expression of TIMP-1 in PF (Figure 3C). All *p* values are presented in Appendix D.

### 2.4. Activity of TGF-β Signaling Pathway in Structural Airway Cells

**Figure 4:**
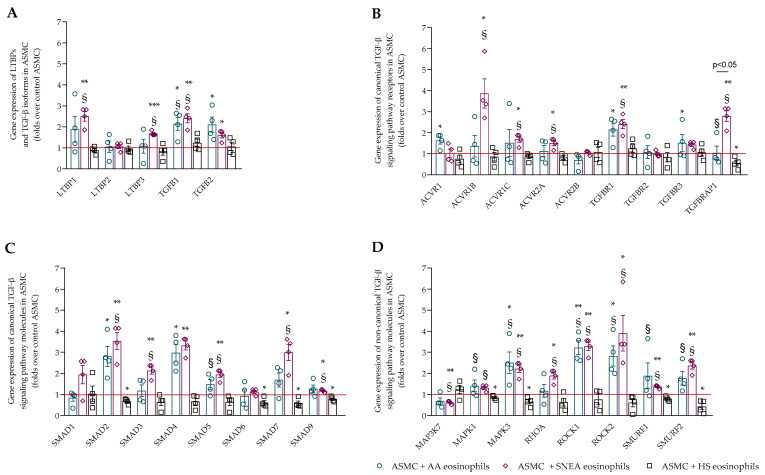
(**A**)—Gene expression of LTBPs and TGF-β isoforms in ASMC; (**B**)—Gene expression of canonical TGF-β signaling pathway receptors in ASMC; (**C**)—Gene expression of canonical TGF-β signaling pathway molecules in ASMC; (**D**)—Gene expression of non-canonical TGF-β signaling pathway molecules in ASMC. AA—allergic asthma; HS—healthy subjects; SNEA—severe non-allergic eosinophilic asthma. AA, *n* = 4, SNEA, *n* = 4, HS, *n* = 4. Data are presented as mean ± SEM, fold change over control ASMC. * *p* <0.05 compared with control ASMC; ** *p* < 0.01 compared with control ASMC; *** *p* < 0.001 compared with control ASMC; § *p* < 0.05 compared with HS eosinophil effect. Statistical analysis between investigated groups—two-sided Mann–Whitney U test (independent data); two-sided Wilcoxon matched-pairs signed-rank test (dependent data); and Wilcoxon signed-rank test, which was used for gene expression analysis against control ASMC that were not incubated with eosinophils.

**Figure 5:**
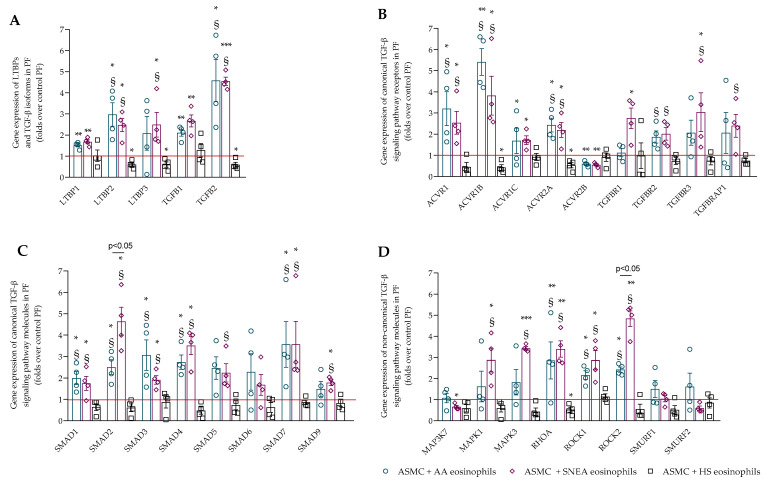
(**A**)—Gene expression of LTBPs and TGF-β isoforms in PF; (**B**)—Gene expression of canonical TGF-β signaling pathway receptors in PF; (**C**)—Gene expression of canonical TGF-β signaling pathway molecules in PF; (**D**)—Gene expression of non-canonical TGF-β signaling pathway molecules in PF. AA—allergic asthma; HS—healthy subjects; SNEA—severe non-allergic eosinophilic asthma. AA, *n* = 4, SNEA, *n* = 4, HS, *n* = 4. Data are presented as mean ± SEM, fold change over control PF. * *p* <0.05 compared with control PF; ** *p* < 0.01 compared with control PF; *** *p* < 0.001 compared with control PF; § *p* < 0.05 compared with HS eosinophil effect. Statistical analysis between investigated groups—two-sided Mann–Whitney U test (independent data); two-sided Wilcoxon matched-pairs signed-rank test (dependent data); and Wilcoxon signed-rank test, which was used for gene expression analysis against control PF that were not incubated with eosinophils.

The effect of asthmatic eosinophils on the gene expression of TGF-β signaling pathway components was evaluated in ASMC and PF. Firstly, the gene expression of latent transforming growth factor-β binding proteins (LTBPs) and the three main isoforms of TGF-β were evaluated in ASMC and PF after incubation with eosinophils. LTBPs are molecules that accumulate TGF-β in ECM. The AA and SNEA eosinophils significantly increased the gene expression of TGF-β1 and TGF-β2 in ASMC compared with control ASMC, while SNEA eosinophils also significantly promoted the gene expression of LTBP1 and LTBP3 in ASMC compared with control ASMC (Figure 4A). The AA eosinophils significantly increased TGF-β1, while SNEA eosinophils increased LTBP1, LTBP3, and TGF-β1 gene expression in ASMC compared with the effect of HS eosinophils. The gene expression of LTBP1, LTBP2, TGF-β1, and TGF-β2 was significantly increased in PF after incubation with AA and SNEA eosinophils compared with control PF (Figure 5A). Additionally, the SNEA eosinophils significantly promoted the gene expression of LTBP3 in PF compared with control PF. Furthermore, the gene expression of TGF-β2 was significantly higher after incubation with AA and SNEA eosinophils, and the SNEA eosinophils had a more pronounced effect on LTBP2 and LTBP3 gene expression in PF, compared with HS eosinophils. TGF-β3 gene expression was absent in all experiments.

Another group of molecules responsible for TGF-β signaling pathway activity is receptors such as activins and TGFB receptors. The AA eosinophils significantly increased ACVR1, TGFBR1, and TGFBR3 gene expression in ASMC, and ACVR1, ACVR1B, ACVR1C, and ACVR2A gene expression in PF compared with control cells (Figure 4B and Figure 5B). SNEA eosinophils significantly increased ACVR1B, ACVR1C, ACVR2A, TGFBR1, and TGFBRAP1 gene expression in ASMC, and ACVR1, ACVR1B, ACVR1C, ACVR2A, TGFBR1, and TGFBR3 in PF compared with control cells. Furthermore, the AA eosinophil effect was significantly higher in terms of TGFBRAP1 gene expression in ASMC, and ACVR1, ACVR1B, ACVR2A, and TGFBR2 gene expression in PF compared with the HS eosinophil effect. Additionally, the SNEA eosinophils significantly promoted the gene expression of ACVR1B, ACVR1C, ACVR2A, TGFBR1, TGFBRAP1 in ASMC, and ACVR1, ACVR1B, ACVR2A, TGFBR2, TGFBR3, and TGFBRAP1 in PF compared with HS eosinophils. The SNEA eosinophils had a greater effect on TGFBRAP1 gene expression than AA eosinophils. However, the ACVR2B gene expression was downregulated by AA and SNEA eosinophils in PF compared with control PF.

The main canonical TGF-β signaling pathway molecules are Smads, which are critically important for cell development and growth. The TGF-β/Smad2/3 pathway acts as a profibrotic agent, while TGF-β/Smad1/5/9 acts as an antifibrotic agent. Our study showed that AA eosinophils significantly increased the gene expression of Smad2 and Smad4 in ASMC, and Smad1, Smad2, Smad3, Smad4, and Smad7 in PF compared with control cells (Figure 4C and Figure 5C). SNEA eosinophils significantly promoted the gene expression of Smad2, Smad3, Smad4, Smad5, Smad7, and Smad9 in ASMC, and Smad1, Smad2, Smad3, Smad4, and Smad7 in PF compared with control cells. Furthermore, the AA eosinophils had a significantly greater effect on the promotion of the gene expression of Smad5 in ASMC and Smad1, Smad2, Smad3, Smad4, and Smad7 in PF compared with HS eosinophils, while SNEA eosinophils significantly promoted Smad2, Smad3, Smad5, and Smad7 gene expression in ASMC and Smad1, Smad2, Smad3, Smad4, Smad5, and Smad7 in PF compared with HS eosinophils. SNEA eosinophils significantly promoted Smad2 gene expression in PF compared with AA eosinophils.

The non-canonical TGF-β signaling pathway consists of various branches of MAPK, Rho-like GTPase, and AKT signaling pathways. The AA eosinophils significantly increased the gene expression of MAPK3, ROCK1, and ROCK2 in ASMC, and RHOA, ROCK1, and ROCK2 in PF compared with control cells, while SNEA eosinophils significantly promoted the gene expression of MAPK3, RHOA, ROCK1, ROCK2, Smurf1, and Smurf2 in ASMC and MAPK1, MAPK3, RHOA, ROCK1, and ROCK2 in PF compared with control cells (Figure 4D and Figure 5D). Furthermore, the gene expression of MAPK1, MAPK3, ROCK1, ROCK2, Smurf1, and Smurf2 in ASMC and RHOA, ROCK1, and ROCK2 in PF was significantly higher after incubation with AA eosinophils compared with HS eosinophils. SNEA eosinophils significantly increased MAPK1, MAPK3, RHOA, ROCK1, and ROCK2 gene expression in ASMC and PF, and Smurf1 and Smurf2 only in PF compared with HS eosinophils. Additionally, the ROCK2 gene expression was significantly higher after incubation with SNEA eosinophils compared with AA eosinophils. However, the SNEA eosinophils downregulated the gene expression of MAP3K7 in ASMC and PF compared with control cells, and ASMC compared with the HS eosinophil effect.

**Figure 6:**
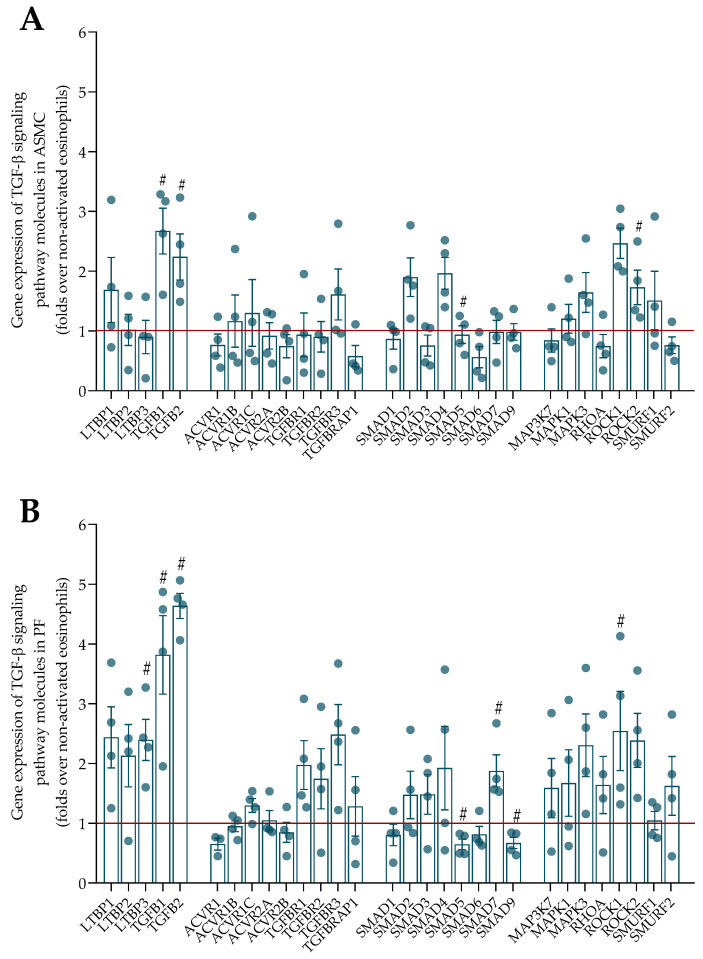
(**A**)—Gene expression of TGF-β signaling pathway molecules in ASMC after incubation with allergen-activated eosinophils; (**B**)—Gene expression of TGF-β signaling pathway molecules in PFafter incubation with allergen-activated eosinophils. AA, *n* = 4, SNEA, *n* = 4, HS, *n* = 4. Data are presented as fold change over the non-activated AA eosinophil effect, mean ± SEM. # *p* < 0.05 compared with non-activated eosinophil effect. Statistical analysis between investigated groups—Wilcoxon signed-rank test was used for gene expression analysis against control ASMC or PF that were not incubated with eosinophils.

Allergen-activated eosinophils significantly increased the gene expression of TGF-β1, TGF-β2, Smad4, and ROCK1 in ASMC, and TGF-β1, TGF-β, LTBP3, and Smad7 in PF, but downregulated Smad5 and Smad9 in PF compared with the non-activated eosinophil effect (Figure 6). All the *p* values are presented in Appendix E.

## 3. Discussion

The study results show that asthmatic eosinophils imbalanced the gene expression of ECM proteins and their homeostasis-regulating components, such as MMPs and TIMPs. The effect of asthmatic eosinophils was more pronounced on the gene expression of collagen I, fibronectin, versican, tenascin C, decorin, vitronectin, periostin, vimentin, MMP-9, ADAM33, TIMP-1, and TIMP-2 gene expression in ASMC, and collagen I, collagen III, fibronectin, elastin, decorin, MMP-2, and TIMP-2 gene expression in PF than HS eosinophils. AA eosinophils had a greater effect on the promotion of the gene expression of the non-canonical TGF-β signaling pathway in ASMC, compared with the effect of SNEA eosinophils on the gene expression of canonical and non-canonical pathway molecules. PF had a more pronounced canonical signaling pathway after incubation with AA and SNEA eosinophils. Allergen-activated eosinophils promoted TGF-β expression in both cell lines.

Type 2 asthma is a chronic eosinophilic airway inflammatory disease characterized by blood and airway eosinophilia. It was shown that eosinophils could adhere to structural airway cells such as ASMC and PF [15,16,17]. Inflammatory processes in the blood and airway cause eosinophils to migrate to the lung. Adhered eosinophils secrete various regulatory mediators, thus promoting ASMC and PF proliferation, migration, and contractility, leading to airway remodeling [18,19]. ASMC and PF are stimulated by mediators secreted by migrated eosinophils, leading to their differentiation to more active cell phenotypes [20]. Accordingly, under TGF-β stimulation, ASMC differentiate to contractile and proliferative–synthetic ASMC phenotypes, and PF to myofibroblasts [21]. The proliferative–synthetic ASMC and myofibroblasts synthesize ECM proteins, and MMPs and TIMPs regulate their homeostasis. However, the homeostasis of ECM proteins is imbalanced in asthma, resulting in ECM deposition in the airway wall. 

Previously, we showed that asthmatic eosinophils increased the gene expression of collagen I and fibronectin [13,19]. In the current study, we evaluated ECM proteins more widely, including the gene expression of MMPs and TIMPs. ECM homeostasis is associated with ECM protein production and degradation. Our study showed that asthmatic eosinophils promote the gene expression of several ECM proteins, such as collagen I, fibronectin, versican, elastin, tenascin C, decorin, periostin, and vimentin in ASMC and PF. The known functions of all studied ECM proteins are presented in Appendix A. Other studies showed that the gene and/or protein expression of collagen I [13,22,23], fibronectin [23,24,25,26,27], versican [28,29], elastin [7], tenascin C [30], periostin [31,32,33,34,35,36,37,38,39], and vimentin [40] was increased in ASMC and PF in asthma studies, including in vivo, ex vivo, in vitro, and animal model studies. Furthermore, the gene expression of the ECM-protein-degrading proteins MMP-2, MMP-9, and ADAM33 in ASMC and PF was increased after incubation with asthmatic eosinophils. Similar results were found in other asthma studies—the MMP-2 and MMP-9 levels were higher in asthmatic airways than in healthy ones [7,41]. It was shown that MMP-2 overexpression protects from asthma by promoting the polarization of macrophages to the M1 phenotype and reducing airway hyperresponsiveness and the expression of Th2 cytokines and IgE [41,42]. The expression of TIMP-1 and TIMP-2 was increased in ASMC and PF under the effect of asthmatic eosinophils. TIMP-1 and TIMP-2 inhibit MMPs, resulting in the increased deposition of non-degraded ECM proteins [43]. 

TGF-β is a pleiotropic cytokine that regulates target cell responses, including apoptosis, survival, proliferation, and differentiation to active cell phenotypes [2,44]. Inflammatory and airway structural cells are capable of the synthesis and secretion of TGF-β, as well as the expression of TGF-β receptors. The effects of TGF-β can be divided into two opposite mechanisms: TGF-β can act as both an anti-inflammatory and pro-inflammatory mediator [45]. As an anti-inflammatory cytokine, TGF-β deactivates macrophages, and as a pro-inflammatory cytokine, it promotes the chemotaxis of eosinophils, T lymphocytes, B lymphocytes, and neutrophils, which induces the proliferation of PF, and suppresses the apoptosis of eosinophils in asthma [2]. TGF-β promotes subepithelial fibrosis by increasing the deposition of ECM proteins as it regulates target gene expression in asthma [46]. The central cells responsible for subepithelial fibrosis are PF, which differentiate into the more active myofibroblasts phenotype and produce ECM proteins under the influence of TGF-β.

Furthermore, TGF-β is responsible for the differentiation of ASMC to more active contractile and synthetic–proliferative phenotypes. The increased TGF-β expression in asthma is associated with airway remodeling. Previously, we showed that TGF-β gene expression was higher in ASMC after incubation with asthmatic eosinophils, and the TGF-β levels in the medium of combined cultures were significantly higher in those incubated with asthmatic eosinophils than with eosinophils isolated from HS [13]. PF and ASMC secrete TGF-β in an inactive form that is deposited in ECM [47]. Inactivated TGF-β latency associated with peptide-1 must be removed to release the active peptide. The reaction is catalyzed by several proteases, including MMP-2 and MMP-9, as well as plasmin, thrombospondin-1, calpanins, and various mediators [48,49,50,51]. 

The canonical or Smad-dependent TGF-β signaling pathway is responsible for increased ECM deposition in the airway wall, while the non-canonical TGF-β signaling pathway is associated with increased contractility, migration, and proliferation [52]. TGF-β phosphorylates its receptors and starts the signaling pathway. Canonical and non-canonical pathways are activated through three TGF-β receptors—TGF-βR1, TGF-βR2, and TGF-βR3. We found that the gene expression of TGF-βR3 was absent in both the ASMC and PF cell lines. It was shown that the expression of TGF-βR1 and TGF-βR2 receptors on the cell’s outer membrane was increased in asthma [53,54]. Then, when the receptors are phosphorylated, the Smad signaling pathway is activated—the phosphorylated Smad2/3 molecules bind to Smad4, which initiates target gene expression, resulting in increased cell proliferation, ECM expression, cell survival, and the promotion of cell proliferation, inflammation, etc. [55]. The non-canonical TGF-β signaling pathway is associated with several other signaling pathways, such as Ras/ERK, JNK, RhoA/ROCK, and MAP3K7 [55]. Our study found that eosinophils activate the gene expression of both the canonical and non-canonical TGF-β signaling pathway components. The study by Wnuk et al. showed that the expression and activation of Smad2 and Smad3 were increased in primary bronchial fibroblasts isolated from asthma patients compared with non-asthma patients [56]. In our study, the profibrotic Smad2 and Smad3 gene expression in PF was higher after incubation with asthmatic eosinophils than HS eosinophils, matching other authors’ findings. However, our study showed the increased gene expression of antifibrotic Smad1 and Smad5 in PF, while in the study by Wnuk et al. the gene expression of Smad1 and Smad5 was downregulated. We propose that differences in the antifibrotic Smad1 and Smad5 gene expression may be due to the treatment used. Schwartze et al. showed that glucocorticosteroids potentiated TGF-β signaling by the Activin receptors and Smad1, Smad5, and Smad9, and blunted signaling by the TGFBR1, Smad2, and Smad3 axis [57]. Another study showed that TGF-β1-neutralizing antibody therapy inhibited TGF-β1 expression and Smad2/3 signaling in nasal and lung tissues in mice [58]. All possible therapies involving the effect of eosinophils and the TGF-β signaling pathway on structural lung cells should be considered. 

Furthermore, the SNEA eosinophils had a more significant effect on these changes, which may be explained by the activation status of the eosinophils. The SNEA eosinophils are more activated compared with eosinophils from mild asthma patients, as the production of various mediators and the expression of cell surface molecules are more pronounced [59,60]. Flood-Page et al. showed that anti-IL-5 therapy significantly reduced tenascin and procollagen III expression in vivo, which could be a promising therapy for severe eosinophilic asthma patients [61]. This effect of anti-IL-5 therapy could be explained by the reduced eosinophil count and activity [62,63,64]. Additionally, we found that the gene expression of TGF-β1 and β2 isoforms, as well as receptors of TGF-β, is more pronounced in ASMC and PF after incubation with asthmatic eosinophils than with HS eosinophils. The elevated levels of TGF-β and its receptors create the “vicious circle”: eosinophil-secreted TGF-β binds to its receptors on the surface of ASMC and PF, thus promoting the expression of TGF-β signaling pathway components, ECM proteins, MMPs, and TIMPs, further promoting inflammatory processes in asthma [2,46,53,65]. 

The exposure to inhaled allergens starts a cascade of processes that results in eosinophil activation and migration to the airways [14]. Several molecules, including eotaxin, IL-4, IL-5, and IL-13, are secreted by Th2 cells and epithelial cells, which promotes eosinophil maturation in bone marrow, their activation in blood, and extravasation in the airways [66,67]. The allergen-activated eosinophils were shown to be more activated [60,68]. Furthermore, we previously showed that allergen-activated eosinophils increased the gene expression of collagen I and fibronectin in ASMC and PF and promoted their migration and ability to contract the collagen gel [19]. In the current study, we showed that allergen-activated eosinophils promoted the gene expression of collagen I, fibronectin, elastin, MMP-12, ADAM33, TGF-β1, TGF-β2, Smad4, and ROCK1 in ASMC, and collagen I, fibronectin, elastin, versican, TIMP-1, TGF-β1, TGF-β2, LTBP3, Smad5, Smad7, and Smad9 in PF. The increased gene expression of the main ECM proteins—collagen I and fibronectin—and TGF-β1 in ASCM and PF was confirmed in our previous studies [13,15,18,19,69]. We claim that allergen-activated eosinophils significantly enhance airway remodeling during asthma.

A possible limitation of our study is that we evaluated the changes in gene expression, but not at the protein level. It is stated that the quantity of transcripts may not always correlate with the protein level. However, it was shown that differentially expressed mRNA correlates significantly better with their protein product than non-differentially expressed mRNA [70]. Under different conditions, the changes in the mRNA levels correlate with the levels of proteins, for example, comparing disease-affected patients with HS. Our gene expression data have no conflict with other authors’ data, as shown in Appendix A and Appendix B, thus suggesting that this in vitro model can help understand the pathogenesis of asthma in vivo. Furthermore, Mathur et al. shown that degranulation of eosinophils isolated from younger asthma patients were higher than from older asthma patients [71]. Other study showed that young donors’ eosinophils potentially had a rejuvenating effect, the aged host thus decreasing local and systemic inflammation and increasing physical and immune fitness in mice [72]. In our study, the SNEA patients were older than AA patients and HS group as SNEA has late-onset manifestation. However, we claim that the age differences did not affect our data as study relied on the severity of the disease but not on the age groups. Previously we showed that SNEA eosinophils had higher gene expression of IL-3Rα, IL-5Rα, GM-CSFRα, and α4, β1, αM integrin subunits than AA eosinophils showing that SNEA eosinophils were more activated [73]. In addition, our other study showed that viability of SNEA eosinophils were higher than AA eosinophils [18]. Other authors also confirmed that eosinophils from severe eosinophilic asthma patients were more active than those from mild and moderate asthma patients [74,75,76]. To conclude, asthmatic eosinophils, particularly from the SNEA patients, altered the gene expression of ECM proteins, MMPs, TIMPs, and molecules of the canonical and non-canonical TGF-β signaling pathway in ASMC and PF. The present in vitro data confirm that eosinophils change the ECM homeostasis, and the suppression of the activity of the TGF-β signaling pathway may be a target to decrease airway remodeling in asthma. 

## 4. Materials and Methods

The research protocol was approved by the Kaunas Regional Biomedical Research Ethics Committee of the Lithuanian University of Health Sciences with permission no. BE-2-13. The research study was registered in the US National Institutes of Health trial registry ClinicalTrials.gov with identifier NCT03388359. 

### 4.1. Study Subjects

The study group consisted of 14 allergic asthma (AA) patients, 9 severe non-allergic eosinophilic asthma (SNEA), and 11 healthy subjects (HS). All subjects were aged between 18 and 80 years. AA and SNEA patients were recruited at the Department of Pulmonology at the Hospital of Lithuanian University of Health Sciences Kauno klinikos. All the study subjects gave written informed consent to participate in the study. At the recruitment stage, all the subjects were screened, and underwent a history and physical examination, spirometry, methacholine challenge test, skin prick test, and complete blood count analysis. 

The applied inclusion and exclusion criteria for all groups are presented in Table 2. 

Inclusion and exclusion criteria were checked at the screening visit. Then, study subjects signed an informed consent form. Spirometry was then performed for all study groups. The methacholine challenge and skin prick test were performed for AA and HS study groups. In addition, during the baseline visit, blood samples were collected, and a bronchial allergen challenge with *D. pteronyssinus* was performed for the AA study group. Twenty-four hours after the bronchial allergen challenge, the second study visit was scheduled for AA patients, and blood samples were re-taken. For SNEA patients and HS groups, only one visit was scheduled, during which the blood samples were collected.

### 4.2. Study Design

The eosinophils were isolated from subjects’ peripheral blood samples using high-density centrifugation and magnetic separation. We used subjects’ eosinophils to evaluate their effect on the gene expression of the ECM proteins, MMPs, and TIMPs, as well as TGF-β signaling pathway molecules in both cell lines. 

**Figure 7:**
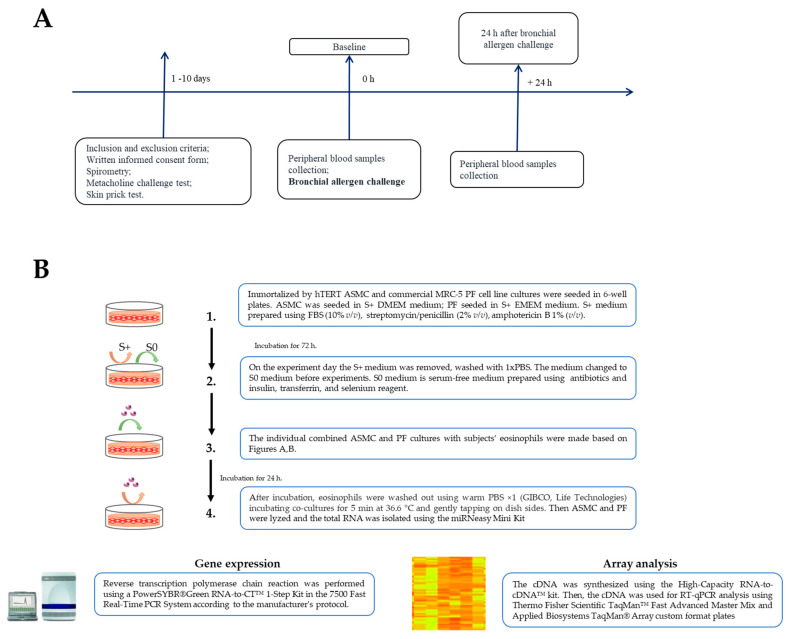
(**A**)―The flowchart of the study design: recruitment of study subjects and clinical examination; (**B**)―The experimental workflow.

A flow chart of the study design and experimental workflow and a detailed experimental plan is presented in Figure 7. 

### 4.3. Lung Function Testing

The lung function of study subjects was evaluated according to baseline forced expiratory volume in 1 s (FEV1), forced vital capacity (FVC), and FEV1/FVC ratio using a Ganshorn spirometer (Ganshorn Medizin Electronic, Niederlauer, Germany). Baseline FEV1, FVC, and FEV1/FVC ratios were recorded as the highest result of three reproducible measurements compared with the predicted values matched for body height, age, and sex using standardized methodology. Each of the values was repeatedly measured at least three times, but no more than eight times and the highest value of FEV1 was taken for analysis.

### 4.4. Measurement of Airway Responsiveness to Methacholine

AA and HS study group subjects underwent measurement of airway responsiveness to methacholine. The inhaled methacholine test was performed using a ProvoX pressure dosimeter (Ganshorn Medizin Electronic, Niederlauer, Germany). Aerosolized methacholine was inhaled at 2 min intervals, with a starting dose of 0.0101 mg. Then, the dose was increased by steps up to 0.121, 0.511, and 1.31 mg cumulative dose until the total cumulative dose was achieved or a 20% decrease in FEV1 was seen from the baseline. The provocative methacholine dose causing a ≥20% fall in FEV1 (PD_20M_) was calculated using the logarithmic dose–response curve by the linear interpolation of the two adjacent data points.

### 4.5. Skin Prick Test

The skin prick test was conducted using standardized allergen extracts from Stallergenes (S.A., Antony, France) for the following allergens: *D. pteronyssinus*, *D. farinae*, birch pollen, and five mixed grass pollens. The histamine hydrochloride (10 mg/mL) was used as a positive control, and the negative control was diluent (saline). The skin prick test was evaluated after 15 min of application. The test results were considered positive if the wheel diameter was at least 3 mm. Only AA patients sensitized to *D. pteronyssinus* were included in the study.

### 4.6. Bronchial Allergen Challenge

All study subjects from the AA group underwent bronchial allergen challenge with *D. pteronyssinus* allergen (Stallergenes S.A.). The bronchoconstricting effect of nebulized saline was first assessed. The aerosolized allergen was inhaled at 10 min intervals starting with a 0.1 histamine equivalent prick (HEP)/mL allergen concentration, increased sequentially to 1.0, 10.0, 20.0, 40.0, and 60.0 HEP/mL until a 20% decrease in FEV1 from the baseline was achieved. 

### 4.7. Eosinophil Isolation and Combined Cell Cultures

Peripheral blood from each study subject was collected in vacutainers with dipotassium ethylenediaminetetraacetic acid (K2EDTA) (BD Vacutainer^®^, Becton Dickinson UK Ltd., Wokingham, UK) before and 24 h after bronchial allergen challenge from AA, and at the baseline visit from SNEA patients and HS. A UniCel^®^ DxH 800 Coulter^®^ Cellular Analysis System automated hematology analyzer (Beckman Coulter, Miami, FL, USA) was used for the complete blood count test. The detailed procedure for eosinophil isolation was taken from previously described procedures [18]. 

Individual combined cell cultures of eosinophils and ASMC or PF were prepared for experiments. ASMC were immortalized by the stable expression of human telomerase reverse transcriptase (hTERT), and the commercial MRC-5 cell line (Sigma, Ronkonkoma, NY, USA) when PF was used. The cell lines were renewed every 6 passages, avoiding the errors related to possible activity and viability differences and cellular senescence. ASMC and PF were grown to 90–95% confluence in medium supplemented with 10% FBS for 72 h. Then, cells were serum deprived before gene expression experiments, ensuring that the cells were in the growth arrest phase, equalizing all cells into the same cell cycle phase, and minimizing the possible influence of proliferation. Isolated eosinophils were used to create the combined cultures with ASMC or PF for 24 h. The ASMC and PF were cultivated in dishes with approximately 2 × 10^5^ cells, and combined cultures were made by adding 5 × 10^4^ of isolated viable eosinophil suspension in the medium of the ASMC or PF. Each experiment was normalized using the control ASMC and PF cell culture that was not incubated with eosinophils. An inverted microscope (CETI Inverso TC100, Medline Scientific, Oxford, UK) was used for cell growth observation and visualization.

### 4.8. RNA Isolation and Quantitative Real-Time PCR Analysis

Eosinophils were separated from ASMC and PF after 24 h of incubation by repeated washing with warm PBS and gentle taps on the plate to remove residual eosinophils. Then, ASMC and PF were used for gene expression analysis. ASMC and PF cells were lysed using TRIzol™ Reagent (Invitro-gen™, Life Technologies, CA, USA), and the total ribonucleic acid (RNA) was isolated using the miRNeasy Mini Kit (Qiagen, Valencia, CA, USA) according to the manufacturer’s instructions. Reverse transcription polymerase chain reaction (RT-PCR) was performed using a PowerSYBR^®^ Green RNA-to-CT™ 1-Step Kit (Applied Biosystems, Foster City, CA, USA) in the 7500 Fast Real-Time PCR System according to the manufacturer’s protocol. AA, SNEA, and HS eosinophils’ effects on gene expression in ASMC and PF cells were evaluated as fold change over the control cells. The gene expression changes were evaluated by fold change from baseline (before allergen challenge) regarding the bronchial allergen effect. The endogenous control of 18S gene expression was used.

The primers used in gene expression analysis are shown in Table 3.

### 4.9. TaqMan Array Analysis of TGF-β Signaling Pathway 

The analysis of the literature sources was used to select the most crucial TGF-β signaling pathway and pathway-related proteins, and the gene expression of selected targets was evaluated in ASMC and PF after incubation with eosinophils using Applied Biosystems TaqMan^®^ Array custom format plates. A total of 32 target genes and 4 endogenous controls (18S, GADPH, GUSB, HPRT1) were selected. A list of target genes and information about their functions is provided in Appendix B. 

Part of the extracted RNA was used to synthesize cDNA using the Thermo Fisher Scientific High-Capacity RNA-to-cDNA™ kit. Then, the cDNA was used for RT-qPCR analysis using Thermo Fisher Scientific TaqMan™ Fast Advanced Master Mix and Applied Biosystems TaqMan^®^ Array custom format plates. 

### 4.10. Statistical Analysis

Statistical analysis was performed using GraphPad Prism 8 for Windows (Version 8.01, 2019; GraphPad Software, Inc., San Diego, CA, USA). The Shapiro–Wilk test was used to confirm the normality assumption of the data distribution. The gene expression data were not distributed normally. Non-parametric tests were used because of the small sample size. The Mann–Whitney U test was used to evaluate the difference between two independent groups, and the Wilcoxon matched-pairs signed-rank test was used to evaluate the difference between two dependent groups. The Wilcoxon signed-rank test was used for gene expression analysis against the control of ASMC or PF cells. Data are presented as the mean and standard error of the mean (SEM) or standard deviation (SD). A value of *p* < 0.05 was considered statistically significant.

## Figures and Tables

**Table 1:** Demographic and clinical characteristics of the study population.

	AA Patients, *n* = 14		SNEA Patients, *n* = 9	HS, *n* = 11
Age, median (range), years	26 (19–47)		48 (28–80) *#	25 (23–46)
Sex, (male/female), *n*	6/8		4/5	5/6
BMI, median (range), kg/m^2^	24 (17–40)		24 (21–38)	22 (17–30)
Sensitization to *D. pteronyssinus*/*D. farinae*/birch/5-grass mixture allergen, *n*	14/11/6/4		NR	NR
Wheel diameter by *D. pteronyssinus*, median (range), mm	7.4 (4.0–15.0)		0	0
PD_20M_, geometric mean (range), mg	0.10 (0.03–0.26)		ND	NR
FEV_1_, L	3.8 ± 0.8		1.8 ± 1.3 *#	4.1 ± 0.8
FEV_1_, % of predicted	94.0 ± 12.0 *		58.0 ± 26.0 *#	102.0 ± 8.8
	Baseline	24 h after allergen challenge		
Blood eosinophil count, × 10^9^/L	0.37 ± 0.25 *	0.44 ± 0.05 *	0.69 ± 0.57 *	0.20 ± 0.09
Blood eosinophil count, %	5.5 ± 3.2 *	6.7 ± 0.73 *	11.0 ± 9.0 *	2.9 ± 1.2
IgE, median (range), IU/mL	144 (31–538) *	293 (34–1325) *	108 (21–795) *	32 (3–67)
FeNO, ppb	54.0 ± 7.1 *	68 ± 11.0	45.0 ± 9.9 *	13.0 ± 1.6

Data presented as a median (range), mean ± SD. AA—allergic asthma; FeNO—fractional exhaled nitric oxide; FEV_1_—forced expiratory volume in 1 s; HS—healthy subject; IgE—immunoglobulin E; ND—not done; NR—not responded; PD_20M_—the provocation dose of methacholine causing a 20% decrease in FEV_1_; SNEA—severe non-allergic eosinophilic asthma. * *p* < 0.01 compared with HS group; # *p* < 0.01 compared with AA group. Statistical analysis between investigated groups—two-sided Mann–Whitney U test (independent data); Wilcoxon matched-pairs signed-rank test (dependent data).

**Table 2:** Inclusion and exclusion criteria for the study population.

	AA Patients (*n* = 14)	SNEA Patients (*n* = 9)	HS (*n* = 11)
Inclusion criteria	Asthma symptoms ≥ 1 year A non-severe course of the disease Positive skin prick test to *D. pteronyssinus* Positive methacholine challenge test	Asthma history ≥ 1 year Negative skin prick test Peripheral blood eosinophil count ≥ 0.3 × 10^9^/L High doses of inhaled steroids and long-acting β agonists	No chronic respiratory and other diseases Negative skin prick test Negative methacholine challenge test
Exclusion criteria	Clinically significant allergy symptoms Active airway infection ≤ 1 month prior to study Asthma exacerbation ≤ 1 month prior to study Use of oral steroids ≤ 1 month prior to study Smoking		

AA—allergic asthma; HS—healthy subject; SNEA—severe non-allergic eosinophilic asthma; *D. pteronyssinus*—Dermatophagoides pteronyssinus.

**Table 3:** Primers used for gene expression analysis.

Gene	Forward 5′-3′	Reverse 5′-3′
18S	CGCCGCTAGAGGTGAAATTC	TTGGCAAATGCTTTCGCTC
Collagen I α1	TCGAGGAGGAAATTCCAATG	ACACACGTGCACCTCATCAT
Collagen III	TATCGAACACGCAAGGCTGTGAGA	GGCCAACGTCCACACCAAATTCTT
Collagen V α1	GGCTCCCGAGAGCAACCT	CGGGACACTCACGAACGAA
Fibronectin	AGCCAGCAGATCGAGAACAT	TCTTGTCCTTGGGGTTCTTG
Elastin	GGCCATTCCTGGTGGAGTTCC	AACTGGCTTAAGAGGTTTGCCTCCA
Versican	GATGTGTATTGTTATGTGGATCA	CATCAAATCTGCTATCAGGG
Tenascin C	GAGACATCTGTGGAAGTGGA	CGTACTCAGTGTCAGGCTTC
Decorin	AAATATTGTGCAAGGCCCGG	TTTTGCTGCCTGAGTCATCG
Vitronectin	CCAGAGCTGCTGCACAGACTA	ATCCCCGCGAGTCACTTG
Periostin	TGCCCTGGTTATATGAGAATGGAAG	GATGCCCAGAGTGCCATAAACA
Vimentin	GCAAAGATTCCACTTTGCGT	GAAATTGCAGGAGGAGATGC
MMP-1	CCTAGTCTATTCATAGCTAATCAAGAGGATGT	AGTGGAGGAAAGCTGTGCATAC
MMP-2	GGCCCTGTCACTCCTGAGAT	GGCATCCAGGTTATCGGGGA
MMP-9	GGCCTCCAACCACCACCAC	CGCCCAGAGAAGAAGAAAAGC
MMP-12	TGCTGATGACATACGTGGCA	AGGATTTGGCAAGCGTTGG
ADAM33	GACCTAGAATGGTGTGCCAGA	AGCCTGGCTTGTCACAGAAG
TIMP-1	AGACCTACACTGTTGGCTGTGAG	GACTGGAAGCCCTTTTCAGAG
TIMP-2	ATGCACATCACCCTCTGTGA	CTCTGTGACCCAGTCCATCC

## Data Availability

This article includes all the data presented in this study.

## Abbreviations

AA	Allergic asthma
ADAM33	A disintegrin and metalloproteinase 33
ASMC	Airway smooth muscle cells
BMI	Body mass index
*D. pteronyssinus*	*Dermatophagoides pteronyssinus*
ECM	Extracellular matrix
FeNO	Fractional exhaled nitric oxide
FEV_1_	Forced expiratory volume in 1 s
FVC	Forced vital capacity
HS	Healthy subjects
hTERT	Human telomerase reverse transcriptase
IgE	Immunoglobulin E
IL	Interleukin
MMP	Matrix metalloproteinase
qRT- PCR	Real-time quantitative reverse transcription PCR
PD_20M_	Provocative dose of methacholine causing a 20% drop in FEV1
PF	Pulmonary fibroblasts
SD	Standard deviation
SEM	Standard error of the mean
SNEA	Severe non-allergic eosinophilic asthma
TGF-β	Transforming growth factor
TIMP	Tissue inhibitor of metalloproteinase

## Appendix A

**Table A1:** A brief review of extracellular matrix components, their homeostasis regulatory functions, and sources.

	Function	Source	↑/↓ in Asthma			
			In Vivo	Ex Vivo	In Vitro	Animal Model
**Extracellular matrix and intracellular proteins, proteoglycans, glycoproteins**						
Collagen I	The integral structural component of many organs; strengthens, supports tissues, and gives rigidity and elasticity.	Smooth muscle cells, fibroblasts, epithelial cells.	↑[22,24,27,77,78,79]	↑[22]	↑[13,22,23]	↑[80,81]
Collagen III	Integral structural component of many organs; regulates the formation of type I and II collagen fibrils diameter. Facilitates platelet aggregation and blood clotting.	Smooth muscle cells, fibroblasts.	↑[24,25,26,27] ↓[79]		[23]	-[80]
Collagen V	Regulates the formation of fiber with type I collagen.	Smooth muscle cells, fibroblasts, endothelial and epithelial cells.	↑[25]			↑[80]
Fibronectin	Structural scaffold which regulates tissue organization and ECM composition. Participates in tissue repair and fibrosis. Regulates platelet function and mediates homeostasis. Regulates cell adhesion, growth, migration, and differentiation. Necessary for embryogenesis. Attenuates activation and degranulation of eosinophils via adhesion.	Smooth muscle cells, fibroblasts, alveolar macrophages, hepatocytes, epithelial cells.	↑[24,78,79]	↑[7]	↑[13,23,82,83]	
Elastin	Allows many tissues in the body to resume their shape after stretching or contracting.	Smooth muscle cells, fibroblasts.		↑[7]		↓[81]
Versican	Regulates cell adhesion, migration, proliferation, and cell apoptosis (reduces). Considered an anti-adhesion molecule.	Smooth muscle cells, fibroblasts, leucocytes.	↑[28]	↑[29]		
Tenascin C	Adhesion-modulating protein that inhibits cell adhesion to fibronectin. Regulates cell proliferation, contraction, migration in developmental differentiation, inflammation, and wound healing. Defense against bacterial and viral infections.	Smooth muscle cells, fibroblasts.	↑[30]			
Decorin	Myokine that participates in fibrillogenesis, regulates TGF-β activity, cell cycle, and autophagy, and inhibits angiogenesis.	Smooth muscle cells, fibroblasts, epithelial cells.	-[28]	↓[29]		
Vitronectin	Regulates cell adhesion, migration, and signal transduction. Binds to membrane-bound integrins that anchor cells to the ECM. Stabilizes plasminogen activator inhibitor-1.	Smooth muscle cells, fibroblasts.	↓[84]		[85]	
Periostin	Increases cell survival, invasion, angiogenesis, metastasis, epithelial–mesenchymal transition, tissue remodeling, regulates cell fate, ECM restructuring, tissue remodeling, and supports adhesion and the migration of cytokine-activated eosinophils.	Smooth muscle cells, fibroblasts, epithelial cells.	↑[31]		↑[32,33,34,86]	↑[35,36,37,38,39]
Vimentin	Supports and anchors the position of organelles in the cytosol. Important for cell flexibility.	Smooth muscle cells, fibroblasts.			↑[87,88]	
**Proteases (metalloendopeptidases)**						
MMP-1	Collagenase breaks down the interstitial collagens, types I, II, and III. Regulates normal physiological processes, such as embryonic development, reproduction, and tissue remodeling.	Immune cells, epithelial cells, fibroblasts.	↑[79,89]		↑[90,91]	
MMP-2	Gelatinase A. Degrades type I and IV collagens. Regulates cell migration, signaling, neovascularization, lymphangiogenesis, and ECM remodeling.	Immune cells, endothelial cells, smooth muscle cells, fibroblasts.	-[92] ↑[79]		↑[93]	↓[42]
MMP-9	Gelatinase B. Regulates angiogenesis, neovascularization, wound repair, and ECM remodeling.	Immune cells, epithelial cells, fibroblasts.	-[94] ↑[79,92,95,96,97]	↑[7]	↑[93]	
MMP-12	Macrophage metalloelastase. The enzyme degrades soluble and insoluble elastin.	Immune cells, smooth muscle cells, fibroblasts.	-[94] ↑[98]	↑[7]	↑[93]	↑[99]
ADAM-33	Includes cell activation, proteolysis, adhesion, fusion, and signaling, associated with bronchial hyper responsiveness. Regulates cell–cell and cell–matrix interactions.	Smooth muscle cells, fibroblasts.	↑[27,100]		-[33]	↑[100]
**The tissue inhibitors of metalloproteinases**						
TIMP-1	Inhibits MMP-1, MMP-3, MMP-7, and MMP-9. Regulates MMPs in wound healing and ECM remodeling.	Smooth muscle cells, fibroblasts.	-[92] ↑[3,79,89,101,102]			
TIMP-2	TIMP-2 functions as both an MMP inhibitor and an activator. TIMP-2 inhibits MMP-2.	Smooth muscle cells, fibroblasts.	↑[103]			

“↑” shows upregulation, “↓” shows downregulation, “-”shows no changes.

## Appendix B

**Table A2:** A brief review of the functions and sources of TGF-β signaling pathway molecules.

	ID Specification		Asthma
ACVR1	Hs00153836_m1	Activin A receptor type I (ACVR1) or ALK-2 (activin receptor-like kinase-2). ACVR1 is composed of 2 subunits. BMP forms a complex with ACVR2A/ACVR2B or BMPR2 with ACVR1 that transduces signal, resulting in the activation of SMAD1, SMAD2, SMAD3, and SMAD6.	
ACVR1B	Hs00923299_m1	Activin receptor type-1B or ALK-4.	
ACVR1C	Hs00377065_m1	ACVR1C or ALK-7.	
ACVR2A	Hs00155658_m1	Activin type 2 receptor.	
ACVR2B	Hs00609603_m1	Activin type 2 receptor.	
LTBP1	Hs00386448_m1	Latent-transforming growth factor beta-binding protein 1; target—TGF-β.	-[104]
LTBP2	Hs00166367_m1	Latent-transforming growth factor beta-binding protein 2; target—TGF-β.	↑[105]
LTBP3	Hs00221445_m1	Latent-transforming growth factor beta-binding protein 3; target—TGF-β.	
MAP3K7	Hs00177373_m1	Mitogen-activated protein kinase kinase kinase 7 (MAP3K7), also known as TAK1, controls a variety of cell functions, including transcription regulation and apoptosis. AK1 regulates cell survival not solely through NF-κB. This kinase has also been shown to regulate downstream cytokine expression such as TNF. Interacts with TAB1.	
MAPK1	Hs00177066_m1	Mitogen-activated protein kinase 1, also known as MAPK1, p42MAPK, and ERK2, are involved in various cellular processes such as proliferation, differentiation, transcription regulation, and development.	-[106]; ↑[107]
MAPK3	Hs00385075_m1	Mitogen-activated protein kinase 3, also known as p44MAPK and ERK1, acts in a signaling cascade that regulates various cellular processes such as proliferation, differentiation, and cell cycle progression in response to various extracellular signals.	↑[107]
RHOA	Hs00357608_m1	Transforming protein RhoA, also known as Ras homolog family member A (RhoA), is primarily associated with cytoskeleton regulation, mostly actin stress fibers formation, actomyosin contractility, and cell development.	↑[108]
ROCK1	Hs00178463_m1	ROCK1 is a protein serine/threonine kinase also known as Rho-associated, coiled-coil-containing protein kinase 1 (ROCK1), plays a role in cancer and, in particular, cell motility, metastasis, and angiogenesis.	↑[109]
ROCK2	Hs00178154_m1	Rho-associated coiled-coil-containing protein kinase 2 regulates cytokinesis, smooth muscle contraction, the formation of actin stress fibers and focal adhesions, and the activation of the c-fos serum response element.	↑[109]
SMAD1	Hs00195432_m1	Involved in direct signaling from the TGF-β receptors and in various biological activities, including cell growth, apoptosis, morphogenesis, development, and immune responses. This protein targets SMAD-specific E3 ubiquitin ligases, such as SMURF1 and SMURF2, and undergoes ubiquitination and proteasome-mediated degradation.	↓[56]
SMAD2	Hs00183425_m1	Involved in direct signaling from the TGF-β receptor. Regulates multiple cellular processes, such as cell proliferation, apoptosis, and differentiation.	↑[3,56,106,110]
SMAD3	Hs00232222_m1	It is involved in direct signaling from the TGF-β receptor. The expression of SMAD3 has been related to the mitogen-activated protein kinase (MAPK/ERK pathway), particularly to the activity of mitogen-activated protein kinase kinase-1 (MEK1). The genes regulated by SMAD3-mediated TGF-β signaling affect differentiation, growth, and death.	↑[56,106,110]
SMAD4	Hs00232068_m1	Role of partnering with R-Smads to recruit co-regulators to the complex interactions with R-Smads, such as SMAD2, SMAD3, SMAD1, SMAD5, and SMAD8 (also called SMAD9) to form heterotrimeric complexes. SMAD4 is a substrate of the Erk/MAPK kinase and GSK3.	
SMAD5	Hs00195437_m1	Involved in direct signaling from the TGF-β receptors involved in cell signaling and modulates signals of bone morphogenetic proteins (BMPs).	↓[56]
SMAD6	Hs00178579_m1	I-Smads that work to suppress the activity of R-Smads associate more specifically with BMP signaling. Interacts with MAP3K7 and Smad7.	↑[104]
SMAD7	Hs00178696_m1	I-Smads that work to suppress the activity of R-Smads, TGF-β signal inhibitor, blocks TGF-β1 and activin associating with the receptor, blocking access to SMAD2. It is an inhibitory SMAD (I-SMAD) and is enhanced by SMURF2.	↓[111]
SMAD9	Hs00195441_m1	Involved in direct signaling from the TGF-β receptor, SMAD9 is involved in cell signaling. When a bone morphogenetic protein binds to a receptor (BMP type 1 receptor kinase), it causes SMAD9 to interact with the SMAD anchor for receptor activation (SARA).	
SMURF1	Hs00410929_m1	E3 ubiquitin-protein ligase SMURF1 is specific for receptor-regulated SMAD proteins in the bone morphogenetic protein (BMP) pathway.	
SMURF2	Hs00224203_m1	E3 ubiquitin-protein ligase SMURF2.	↑[56]
TGFB1	Hs00234244_m1	Transforming growth factor-beta 1. It also acts as a negative autocrine growth factor. Dysregulation of TGF-β activation and signaling may result in apoptosis. Many cells synthesize TGF-β, and almost all of them have specific receptors for this peptide. Interacts with LTBP1, decorin, and TGFBR1.	↑[106]
TGFB2	Hs00234245_m1	Transforming growth factor-beta 2 is known to suppress the effects of interleukin-dependent T-cell tumors.	↑[3]
TGFB3	Hs00610319_m1	Transforming growth factor-beta 3 is involved in cell differentiation, embryogenesis, and development. TGF-β3 also plays an essential role in controlling the development of lungs in mammals by regulating cell adhesion and ECM formation in this tissue and controlling wound healing by regulating the movements of epidermal and dermal cells in injured skin. Interacts with TGFBR2.	
TGFBR1	Hs00559661_m1	Transforming growth factor-beta receptor I (activin A receptor type II-like kinase). The protein encoded by this gene forms a heteromeric complex with type II TGF-β receptors when bound to TGF-β, transducing the TGF-β signal from the cell surface to the cytoplasm.	-[3]
TGFBR2	Hs00234257_m1	Transforming growth factor, beta receptor II.	-[3]
TGFBR3	Hs00188614_m1	Betaglycan, also known as transforming growth factor-beta receptor III (TGFBR3), is a cell-surface chondroitin sulfate. It is not involved directly in TGF-β signal transduction, but by binding to various members of the TGF-β superfamily at the cell surface, it acts as a reservoir of ligands for TGF-β receptors.	
TGFBRAP1	Hs00174128_m1	Transforming growth factor-beta receptor-associated protein 1 (TRAP1). It is associated with inactive heteromeric TGF-β and activin receptor complexes, mainly through the type II receptor, and is released upon signaling activation. May recruit SMAD4 to the vicinity of the receptor complex and facilitate its interaction with receptor-regulated Smads, such as SMAD2.	

“↑” shows upregulation, “↓” shows downregulation, “-”shows no changes.

## Appendix C

**Table A3:** The results of ECM gene expression analysis and significant changes in gene expression levels.

	AA, mean ± SEM	AA 24 h after BAC, mean ± SEM	SNEA, mean ± SEM	HS, mean ± SEM	AA Compared with HS, *p =*	SNEA Compared with HS, *p* =	AA 24 h after BAC Compared with Baseline Result, *p* =	AA Compared with SNEA, *p* =
**ASMC**								
Collagen I	1.9 ± 0.2	1.8 ± 0.2	3.2 ± 0.6	0.9 ± 0.1	**0.0002**	**<0.0001**	**0.0002**	**0.0425**
Collagen III	1.5 ± 0.2	1.0 ± 0.1	1.3 ± 0.2	0.9 ± 0.2	**0.0123**	0.0535	0.5879	0.5230
Collagen V	1.2 ± 0.1	1.0 ± 0.1	1.4 ± 0.2	0.9 ± 0.3	**0.0107**	**0.0200**	0.5747	0.0988
Fibronectin	2.6 ± 0.6	1.4 ± 0.1	3.8 ± 0.6	1.0 ± 0.2	**0.0107**	**<0.0001**	**0.0002**	**0.0202**
Elastin	1.4 ± 0.2	1.5 ± 0.2	0.9 ± 0.2	1.0 ± 0.1	0.3607	>0.9999	**0.0105**	0.3575
Versican	1.5 ± 0.2	1.1 ± 0.1	2.1 ± 0.6	1.3 ± 0.2	0.5691	0.5027	0.4973	0.6948
Tenascin C	1.4 ± 0.2	0.8 ± 0.2	1.4 ± 0.2	1.3 ± 0.1	0.6490	>0.9999	0.2439	0.7809
Decorin	2.4 ± 0.6	1.1 ± 0.1	3.3 ± 0.7	1.0 ± 0.3	**0.0154**	**0.0057**	0.3054	0.2349
Vitronectin	2.2 ± 0.6	1.1 ± 0.1	3.1 ± 0.8	0.7 ± 0.2	**0.0410**	**0.0031**	0.4861	0.2414
Periostin	1.7 ± 0.3	1.3 ± 0.3	1.3 ± 0.1	1.1 ± 0.1	0.1056	0.6556	0.4548	0.1444
Vimentin	1.9 ± 0.3	1.1 ± 0.2	2.4 ± 0.6	1.2 ± 0.1	0.1500	0.1690	0.7869	0.7810
**PF**								
Collagen I	1.8 ± 0.3	1.9 ± 0.3	2,5 ± 0,4	1.3 ± 0.2	0.3031	**0.0159**	**0.0266**	0.1858
Collagen III	2.8 ± 0.5	1.0 ± 0.2	3,9 ± 0,8	0.9 ± 0.1	0.6490	**<0.0001**	0.4143	0.4310
Collagen V	1.1 ± 0.2	0.7 ± 0.1	0,7 ± 0,1	1.5 ± 0.4	0.6490	0.0804	0.0579	**0.0145**
Fibronectin	2.7 ± 0.5	2.3 ± 0.3	3.2 ± 0.7	1.3 ± 0.2	0.1191	0.0562	**0.0002**	0.9479
Elastin	2.4 ± 0.3	1.3 ± 0.2	2.7 ± 0.4	0.9 ± 0.2	**0.0001**	**0.0003**	0.0803	0.6948
Versican	1.5 ± 0.2	1.3 ± 0.1	1.1 ± 0.2	0.8 ± 0.2	**0.0107**	0.1519	**0.0061**	0.2349
Tenascin C	0.8 ± 0.2	0.9 ± 0.1	0.6 ± 0.2	1.3 ± 0.1	**0.0218**	**0.0251**	0.5417	0.4310
Decorin	3.1 ± 0.5	1.2 ± 0.2	1.4 ± 0.4	1.2 ± 0.3	**0.0048**	0.9443	0.7354	**0.0157**
Vitronectin	0.9 ± 0.3	1.4 ± 0.2	0.6 ± 0.1	1.0 ± 0.2	0.5691	0.3702	0.1677	0.8446
Periostin	1.5 ± 0.2	1.3 ± 0.2	1.3 ± 0.2	1.0 ± 0.1	0.1339	0.4561	0.1272	0.6948
Vimentin	1.4 ± 0.3	1.2 ± 0.2	1.4 ± 0.3	1.3 ± 0.2	0.7330	0.9408	0.1909	0.6948

Gene expression is presented as fold change over control ASMC or PF that were not incubated with eosinophils, mean ± SEM. ASMC—airway smooth muscle cell, PF—pulmonary fibroblast. *p* values in bold show significant changes in gene expression. Statistical analysis between investigated groups—two-sided Mann–Whitney U test (independent data); two-sided Wilcoxon matched-pairs signed-rank test (dependent data); and Wilcoxon signed-rank test, which was used for gene expression analysis.

## Appendix D

**Table A4:** The results of MMP and TIMP gene expression analysis and significant changes in gene expression levels.

	AA, mean ± SEM	AA 24 h after BAC, mean ± SEM	SNEA, mean ± SEM	HS, mean ± SEM	AA Compared with HS, *p* =	SNEA Compared with HS, *p* =	AA 24 h after BAC Compared with Baseline Result, *p* =	AA Compared with SNEA, *p* =
**ASMC**								
MMP-1	0.7 ± 0.1	1.3 ± 0.2	0.6 ± 0.2	1.0 ± 0.2	0.2284	0.2610	0.3396	0.7938
MMP-2	1.6 ± 0.3	0.8 ± 0.2	1.6 ± 0.3	1.1 ± 0.3	0.2226	0.1519	0.3575	0.7561
MMP-9	1.4 ± 0.2	1.4 ± 0.3	2.1 ± 0.4	1.1 ± 0.2	0.2767	0.0562	0.2958	0.2093
MMP-12	0.7 ± 0.1	1.8 ± 0.3	0.8 ± 0.2	1.2 ± 0.2	**0.0059**	0.0952	**0.0166**	0.6948
ADAM33	1.6 ± 0.2	1.9 ± 0.4	3.7 ± 0.5	1.6 ± 0.5	0.2962	**0.0042**	**0.0295**	**0.0006**
TIMP-1	1.6 ± 0.2	0.9 ± 0.1	0.9 ± 0.1	1.0 ± 0.2	**0.0129**	**0.0125**	0.8077	0.6470
TIMP-2	1.8 ± 0.3	1.2 ± 0.1	1.2 ± 0.1	1.0 ± 0.2	**0.0352**	**0.0381**	0.1726	>0.9999
**PF**								
MMP-1	0.7 ± 0.1	0.9 ± 0.2	1.5 ± 0.6	1.4 ± 0.2	**0.0184**	0.5027	0.6257	0.6470
MMP-2	1.6 ± 0.2	1.4 ± 0.3	1.9 ± 0.6	1.1 ± 0.3	0.3607	0.1519	0.2958	0.9479
MMP-9	2.0 ± 0.5	1.4 ± 0.2	2.0 ± 0.3	1.2 ± 0.2	0.0780	0.4244	0.1531	0.7303
MMP-12	1.6 ± 0.3	1.2 ± 0.2	0.5 ± 0.1	1.2 ± 0.2	0.4244	**0.0251**	0.5016	**0.0026**
ADAM33	1.4 ± 0.3	1.2 ± 0.2	1.7 ± 0.2	0.9 ± 0.2	0.1191	**0.0031**	0.2958	0.2624
TIMP-1	1.5 ± 0.2	0.7 ± 0.1	1.5 ± 0.3	0.7 ± 0.1	**0.0015**	**0.0251**	**0.0245**	>0.9999
TIMP-2	1.7 ± 0.3	1.1 ± 0.2	1.5 ± 0.2	0.9 ± 0.3	**0.0352**	**0.0465**	0.9032	0.7438

Gene expression is presented as fold change over control ASMC or PF that were not incubated with eosinophils, mean ± SEM. *p* values in bold show significant changes in gene expression. Statistical analysis between investigated groups—two-sided Mann–Whitney U test (independent data); two-sided Wilcoxon matched-pairs signed-rank test (dependent data); and Wilcoxon signed-rank test, which was used for gene expression analysis against control ASMC and PF that were not incubated with eosinophils.

## Appendix E

**Table A5:** *p* values of evaluated gene expression in Figure 5 and Figure 6.

Gene	ASMC							
	AA, mean ± SEM	AA 24 h after BAC, mean ± SEM	SNEA, mean ± SEM	HS, mean ± SEM	AA Compared with HS, *p* =	SNEA Compared with HS, *p* =	AA 24 h after BAC Compared with Baseline Result, *p* =	AA Compared with SNEA, *p* =
**LTBPs and TGF-** **β**								
LTBP1	1.9 ± 0.6	1.7 ± 0.5	2.5 ± 0.2	0.9 ± 0.1	0.2000	**0.0286**	0.2964	0.4286
LTBP2	1.1 ± 0.3	1.0 ± 0.3	1.1 ± 0.1	1.0 ± 0.1	0.6857	0.8286	0.9431	0.8000
LTBP3	1.1 ± 0.3	0.9 ± 0.3	1.7 ± 0.1	0.8 ± 0.2	0.6857	**0.0286**	0.7424	0.3143
TGF-β1	2.2 ± 0.3	2.7 ± 0.4	2.4 ± 0.2	1.2 ± 0.2	**0.0114**	**0.0286**	**0.0222**	0.8857
TGF-β2	2.1 ± 0.4	2.2 ± 0.4	1.6 ± 0.1	1.1 ± 0.2	0.0571	0.1143	**0.0490**	0.4571
TGF-β3	-	-	-	-	-	-	-	-
**Canonical TGF-β signaling pathway receptors**								
ACVR1	1.6 ± 0.2	0.8 ± 0.2	1.0 ± 0.2	0.8 ± 0.2	0.0571	0.4857	0.2935	0.1143
ACVR1B	1.4 ± 0.5	1.2 ± 0.4	3.9 ± 0.7	0.9 ± 0.2	0.6857	**0.0286**	0.7292	0.0571
ACVR1C	1.5 ± 0.6	1.3 ± 0.6	1.7 ± 0.1	0.9 ± 0.1	0.7714	**0.0286**	0.6271	0.4286
ACVR2A	1.1 ± 0.3	0.9 ± 0.2	1.5 ± 0.1	0.8 ± 0.1	0.8857	**0.0286**	0.7374	0.3143
ACVR2B	0.7 ± 0.2	0.8 ± 0.2	1.0 ± 0.0	1.1 ± 0.2	0.6857	>0.9999	0.2870	0.0571
TGFBR1	2.2 ± 0.3	0.9 ± 0.4	2.4 ± 0.2	1.2 ± 0.2	0.1143	**0.0286**	0.8748	0.8857
TGFBR2	1.1 ± 0.3	0.9 ± 0.3	1.0 ± 0.1	0.8 ± 0.2	0.4857	0.6286	0.7302	0.6286
TGFBR3	1.5 ± 0.4	1.6 ± 0.4	1.4 ± 0.1	1.1 ± 0.2	0.6857	0.1143	0.2479	>0.9999
TGFBRAP1	1.1 ± 0.3	0.6 ± 0.2	2.8 ± 0.2	0.6 ± 0.1	0.2286	**0.0286**	0.1003	**0.0286**
**Canonical TGF-β signaling pathway molecules**								
Smad1	0.8 ± 0.2	0.9 ± 0.2	2.0 ± 0.4	1.0 ± 0.4	0.8857	0.1714	0.4872	0.1714
Smad2	2.8 ± 0.5	1.9 ± 0.3	3.5 ± 0.4	0.7 ± 0.1	**0.0286**	**0.0286**	0.0690	0.3143
Smad3	1.2 ± 0.3	0.8 ± 0.2	2.1 ± 0.2	0.6 ± 0.2	0.3143	**0.0286**	0.2640	0.0571
Smad4	3.0 ± 0.4	2.0 ± 0.3	3.3 ± 0.2	0.7 ± 0.2	**0.0286**	**0.0286**	**0.0355**	0.6286
Smad5	1.5 ± 0.2	0.9 ± 0.2	1.9 ± 0.1	0.6 ± 0.2	**0.0286**	**0.0286**	0.7114	0.1714
Smad6	0.9 ± 0.3	0.6 ± 0.2	1.1 ± 0.1	0.6 ± 0.1	0.6857	0.0571	0.0920	0.6286
Smad7	1.7 ± 0.3	1.0 ± 0.2	3.0 ± 0.4	0.6 ± 0.1	0.0571	**0.0286**	0.9456	0.1143
Smad9	1.3 ± 0.2	1.0 ± 0.1	1.2 ± 0.0	0.8 ± 0.1	0.0571	**0.0286**	0.9086	0.8000
**Non-canonical TGF-β signaling pathway molecules**								
MAP3K7	0.7 ± 0.2	0.8 ± 0.2	0.6 ± 0.0	1.2 ± 0.2	0.0571	**0.0286**	0.4732	0.6286
MAPK1	1.4 ± 0.3	1.2 ± 0.2	1.3 ± 0.1	0.9 ± 0.1	**0.0286**	**0.0286**	0.4593	>0.9999
MAPK3	2.5 ± 0.5	1.6 ± 0.3	2.2 ± 0.2	0.7 ± 0.1	**0.0286**	**0.0286**	0.1485	>0.9999
RhoA	1.2 ± 0.3	0.8 ± 0.2	1.9 ± 0.2	0.6 ± 0.2	0.2000	**0.0286**	0.2861	0.1143
ROCK1	3.2 ± 0.3	2.5 ± 0.3	3.3 ± 0.2	0.8 ± 0.2	**0.0286**	**0.0286**	**0.0103**	>0.9999
ROCK2	2.8 ± 0.5	1.7 ± 0.3	3.9 ± 0.8	0.6 ± 0.2	**0.0286**	**0.0286**	0.0858	0.3143
Smurf1	1.9 ± 0.6	1.5 ± 0.5	1.4 ± 0.0	0.8 ± 0.1	**0.0286**	**0.0286**	0.3742	>0.9999
Smurf2	1.8 ± 0.3	0.8 ± 0.1	2.4 ± 0.2	0.4 ± 0.1	**0.0286**	**0.0286**	0.1880	0.2857
PF								
**LTBPs and TGF-β**								
LTBP1	1.9 ± 0.6	2.4 ± 0.5	2.5 ± 0.2	0.9 ± 0.1	0.3429	0.2000	0.0667	0.2000
LTBP2	1.1 ± 0.3	2.1 ± 0.5	1.1 ± 0.1	1.0 ± 0.1	**0.0286**	**0.0286**	0.1189	0.6857
LTBP3	1.1 ± 0.3	2.4 ± 0.3	1.7 ± 0.1	0.8 ± 0.2	0.3429	**0.0286**	**0.0269**	0.6857
TGF-β1	2.2 ± 0.3	3.8 ± 0.7	2.4 ± 0.2	1.2 ± 0.2	0.0571	0.0571	**0.0232**	0.2000
TGF-β2	2.1 ± 0.4	4.6 ± 0.2	1.6 ± 0.1	1.1 ± 0.2	**0.0286**	**0.0286**	**0.0004**	>0.9999
TGF-β3	-	-	-	-	-	-	-	-
**Canonical TGF-β signaling pathway receptors**								
ACVR1	1.6 ± 0.2	0.7 ± 0.1	1.0 ± 0.2	0.8 ± 0.2	**0.0286**	**0.0286**	0.0748	0.6257
ASVR1B	1.4 ± 0.5	1.0 ± 0.1	3.9 ± 0.7	0.9 ± 0.2	**0.0286**	**0.0286**	0.6138	0.2000
ACVR1C	1.5 ± 0.6	1.3 ± 0.1	1.7 ± 0.1	0.9 ± 0.1	0.6857	0.0571	0.0843	0.8857
ACVR2A	1.1 ± 0.3	1.1 ± 0.2	1.5 ± 0.1	0.8 ± 0.1	**0.0286**	**0.0286**	0.7778	0.8857
ACVR2B	0.7 ± 0.2	0.9 ± 0.2	1.0 ± 0.0	1.1 ± 0.2	0.3429	0.3429	0.4362	0.6857
TGFBR1	2.2 ± 0.3	2.0 ± 0.4	2.4 ± 0.2	1.2 ± 0.2	0.6857	0.1143	0.0967	0.0571
TGFBR2	1.1 ± 0.3	1.7 ± 0.5	1.0 ± 0.1	0.8 ± 0.2	**0.0286**	**0.0286**	0.2365	0.8857
TGFBR3	1.5 ± 0.4	2.5 ± 0.5	1.4 ± 0.1	1.1 ± 0.2	0.0571	**0.0286**	0.0605	0.6857
TGFBRAP1	1.1 ± 0.3	1.3 ± 0.5	2.8 ± 0.2	0.6 ± 0.1	0.6857	**0.0286**	0.6079	0.8857
**Canonical TGF-β signaling pathway molecules**								
Smad1	0.8 ± 0.2	0.8 ± 0.2	2.0 ± 0.4	1.0 ± 0.4	**0.0286**	**0.0286**	0.3531	0.6857
Smad2	2.8 ± 0.5	1.5 ± 0.4	3.5 ± 0.4	0.7 ± 0.1	**0.0286**	**0.0286**	0.3172	**0.0286**
Smad3	1.2 ± 0.3	1.5 ± 0.3	2.1 ± 0.2	0.6 ± 0.2	**0.0286**	**0.0286**	0.2421	0.3429
Smad4	3.0 ± 0.4	1.9 ± 0.7	3.3 ± 0.2	0.7 ± 0.2	**0.0286**	**0.0286**	0.2778	0.3429
Smad5	1.5 ± 0.2	0.7 ± 0.1	1.9 ± 0.1	0.6 ± 0.2	0.0571	**0.0286**	**0.0309**	0.6857
Smad6	0.9 ± 0.3	0.8 ± 0.1	1.1 ± 0.1	0.6 ± 0.1	0.1143	0.1143	0.2574	0.8857
Smad7	1.7 ± 0.3	1.9 ± 0.3	3.0 ± 0.4	0.6 ± 0.1	**0.0286**	**0.0286**	**0.0473**	0.8857
Smad9	1.3 ± 0.2	0.7 ± 0.1	1.2 ± 0.0	0.8 ± 0.1	0.2000	**0.0286**	**0.0413**	0.4857
**Non-canonical TGF-β signaling pathway molecules**								
MAP3K7	0.7 ± 0.2	1.6 ± 0.5	0.6 ± 0.0	1.2 ± 0.2	0.1143	>0.9999	0.3193	0.3429
MAPK1	1.4 ± 0.3	1.7 ± 0.6	1.3 ± 0.1	0.9 ± 0.1	0.2000	**0.0286**	0.3138	0.2000
MAPK3	2.5 ± 0.5	2.3 ± 0.5	2.2 ± 0.2	0.7 ± 0.1	0.0571	**0.0286**	0.0877	0.2000
RhoA	1.2 ± 0.3	1.6 ± 0.5	1.9 ± 0.2	0.6 ± 0.2	**0.0286**	**0.0286**	0.2724	0.4857
ROCK1	3.2 ± 0.3	2.5 ± 0.7	3.3 ± 0.2	0.8 ± 0.2	**0.0286**	**0.0286**	**0.0102**	0.3429
ROCK2	2.8 ± 0.5	2.4 ± 0.5	3.9 ± 0.8	0.6 ± 0.2	**0.0286**	**0.0286**	0.0553	**0.0286**
Smurf1	1.9 ± 0.6	1.0 ± 0.2	1.4 ± 0.0	0.8 ± 0.1	0.1143	0.1143	0.7863	0.6857
Smurf2	1.8 ± 0.3	1.6 ± 0.5	2.4 ± 0.2	0.4 ± 0.1	0.4857	0.4857	0.2923	0.1143

Numbers in bold highlight the significant differences between investigated groups. Statistical analysis—Wilcoxon matched-pairs signed-rank test (dependent data), Mann–Whitney U test (independent data), and Wilcoxon signed-rank test (against the HS eosinophil effect or AA before and 24 h after bronchial allergen challenge).
